# Childhood sexual abuse predicts treatment outcome in conversion disorder/functional neurological disorder. An observational longitudinal study

**DOI:** 10.1002/brb3.1558

**Published:** 2020-02-07

**Authors:** Christina M. Van der Feltz‐Cornelis, Sarah F. Allen, Jonna F. Van Eck van der Sluijs

**Affiliations:** ^1^ Clinical Centre of Excellence for Body, Mind and Health GGz Breburg Tilburg The Netherlands; ^2^ Tranzo Department Tilburg University Tilburg The Netherlands; ^3^ Department of Health Sciences HYMS University of York York UK

**Keywords:** adverse childhood experiences, childhood sexual abuse, conversion disorder, functional neurological disorder, predictive factors, treatment outcome

## Abstract

**Objective:**

Explore trauma, stress, and other predictive factors for treatment outcome in conversion disorder/functional neurological disorder (CD/FND).

**Methods:**

Prospective observational design. Clinical cohort study among consecutive outpatients with DSM‐IV CD/FND in a specialized mental health institution for somatic symptom disorders and related disorders (SSRD), presented between 1 February 2010 and 31 December 2017. Patient files were assessed for early childhood trauma, childhood sexual abuse, current stress, and other predictive factors. Patient‐related routine outcome monitoring (PROM) data were evaluated for treatment outcome at physical (Patient Health Questionnaire [PHQ15], Physical Symptoms Questionnaire [PSQ]) level as primary outcome, and depression (Patient Health Questionnaire [PHQ9]), anxiety (General Anxiety Disorder [GAD7]), general functioning (Short Form 36 Health Survey [SF36]), and pain (Brief Pain Inventory [BPI]) as secondary outcome.

**Results:**

A total of 64 outpatients were included in the study. 70.3% of the sample reported childhood trauma and 64.1% a recent life event. Mean scores of patients proceeding to treatment improved. Sexual abuse in childhood (*F*(1, 28) = 30.068, *β* = 0.608 *p *< .001) was significantly associated with worse physical (PHQ15, PSQ) treatment outcome. 42.2% reported comorbid depression, and this was significantly associated with worse concomitant depressive (PHQ9) (*F*[1, 39] = 11.526, *β* = 0.478, *p *= .002) and anxiety (GAD7) (*F*[1,34] = 7.950, *β* = 0.435, *p *= .008) outcome.

**Conclusion:**

Childhood sexual abuse is significantly associated with poor treatment outcome in CD/FND. Randomized clinical trials evaluating treatment models addressing childhood sexual abuse in CD are needed.

## Graphical abstract

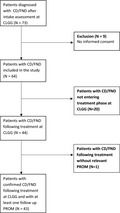

**Significant outcomes**

- Childhood trauma occurs in 70.3% of patients with conversion disorder/FND.
- Childhood sexual abuse is significantly associated with poor treatment outcome for physical symptoms in patients with conversion disorder/FND.
- Randomized clinical trials evaluating treatment models addressing childhood sexual abuse in conversion disorder/FND are needed.

**Limitations**

- The findings of this study are generalizable to chronic complex conversion disorder/FND treated in the specialty mental health setting, not to incident cases.
- Although in comparison with other studies in this field, the sample size of this study can be considered large, it is relatively small compared with other clinical epidemiological cohort studies.

## 1 INTRODUCTION

### 1.1 Background

Conversion disorder (CD) (Association AP, 2013) or functional neurological disorder (FND) (Stone, Hallett, Carson, Bergen, & Shakir, 2014) involves symptoms or deficits affecting voluntary motor or sensory function that suggests a neurologic condition, but lack a neurologic explanation after appropriate neurological examination (Allin, Streeruwitz, & Curtis, 2005; Association AP, 2013; Aybek, Kanaan, & David, 2008). Approximately 20%–30% of patients at neurological clinics suffer from CD/FND (Stone et al., 2014; Aybek et al., 2008; Feinstein, 2018). CD/FND occurs mostly in the 2nd–4th decade and generally takes a protracted course with high limitations in general, social and work functioning in addition to high medical healthcare utilization (Merkler et al., 2016). Family members also feel a burden (Griffith, Polles, & Griffith, 1998) as patients with CD/FND often need long‐time intensive help, can become dependent on a wheelchair, and may need adaptations of their house and living arrangements. As treatment may have relatively little to offer, family members often provide care. Patients with CD/FND visit neurological wards for diagnostic procedures(Stone & Vermeulen, 2016; Vermeulen & Willems, 2015), often with unsatisfactory results(Régny & Cathébras, 2016; Stone & Vermeulen, 2016; Sveinson, Stafánsson, & Hjaltason, 2009; Van der Feltz‐Cornelis, 2015), and there is a high return rate of patients (Merkler et al., 2016). A 3‐year follow‐up study of 42 patients with CD/FND showed persistence in abnormal movements in more than 90% of the patients (Feinstein, Stergiopoulos, Fine, & Lang, 2001). Thus, the individual and societal burden of CD/FND is high (Mace & Trimble, 1996).

The work on unraveling the pathogenesis of CD/FND, and the role of recent and early stress as well as psychological and biological vulnerabilities, is ongoing, as is indicated in a recent review (Keynejad, Kanaan, Pariante, Reuber, & Nicholson, 2018). Cross‐sectional studies exploring cortisol and the stress response in CD/FND found conflicting results. Apazoglou found a baseline HPA axis, and sympathetic hyperarousal state in motor CD/FND was related to life adversities. During a social stress test, dissociation between stress perceived as such by patients with CD/FND, and their biological stress markers, was observed (Apazoglou, Wegrzyk, Frasca Polara, & Aybek, 2017). However, Maurer, LaFaver, Ameli, Toledo, and Hallett (2015) found that current stress levels were not altered in patients with functional movement disorders and suggested that the insistence on heightened stress levels in these patients is unjustified. In an exploration of immune function in CD/FND, Tilyeki et al found temporarily decreased serum TNF‐α levels in the acute phase of CD/FND, suggesting that stress associated with CD/FND might suppress immune function in the acute phase of CD/FND (Tiyekli, Çalıyurt, & Tiyekli, 2013). An association between stress‐related neuroplasticity, CD/FND, and reduced insular volume was identified in an MRI study in patients with FND and childhood adversity (Perez et al., 2017). Childhood physical or sexual abuse has been found in 44% of patients with CD/FND (Roelofs, Spinhoven, Sandijck, Moene, & Hoogduin, 2005) and is associated with higher symptom loads in patients with nonepileptic seizures (Selkirk, Duncan, Oto, & Pelosi, 2008). In a case series study, more than 50% reported a history of exposure to physical violence and 25% reported a history of sexual assault in childhood (Régny & Cathébras, 2016). However, a study exploring the role of trauma and stress in the development of CD/FND could not confirm that these events were always present (Ludwig et al., 2018), and the DSM‐5 classification for CD/FND no longer requires the presence of a stressor (Association AP, 2013).

Longitudinal epidemiological studies that explored patient characteristics related to prognosis found that CD/FND symptoms persisted or recurred in 39%–70% of cases and were associated with a poor quality of life (Group S, 2007; Martlew & Marson, 2014; Régny & Cathébras, 2016). An observational study in the neurological setting found that psychiatric comorbidity and an inability to consider the psychological nature of their condition was associated with a poor longer term prognosis (Feinstein et al., 2001). Short duration of symptoms before start of treatment, early diagnosis, and high satisfaction with care predicted a positive outcome. Delayed diagnosis and comorbid personality disorder predicted a negative outcome (Gelauff, Stone, Edwards, & Carson, 2014; Halligan, Bass, & Marshall, 2001). Depression (38%–50%), anxiety disorders (35%), dissociative disorders (48.3%) (Akyüz, Gökalp, Erdiman, Oflaz, & Karsidag, 2017; Régny & Cathébras, 2016), and pain (50%) (Régny & Cathébras, 2016) were the most commonly reported comorbidities. However, none of these studies, including the study of Ludwig et al. (2018), explored the effect of such factors on treatment outcome in CD/FND.

### 1.2 Rationale

As a correlate of the evidence gap on pathogenesis, treatment of CD/FND has a limited evidence base (Ruddy & House, 2005). Mental health care for chronic CD/FND is often a combination of outpatient and inpatient, multidisciplinary treatment (Demartini et al., 2014), of long duration and with limited results (Carson et al., 2012; Krull, 2014). Although there is some evidence for cognitive behavioral therapy (Dallocchio, Bombieri, Arnó, & Erro, 2016), physiotherapy (Nielsen, Stone, & Edwards, 2013), transcranial magnetic stimulation (Garcin et al., 2013; Parin & Chastan, 2014; Schönfeldt‐Lecuona, Connemann, Spitzer, & Herwig, 2003), and hypnosis (Moene, Spinhoven, Hoogduin, & Dyck, 2002; Moene, Spinhoven, Hoogduin, & Dyck, 2003), there is little reliable evidence to support the use of any treatment, including CBT (Martlew & Marson, 2014; Ruddy & House, 2005). These knowledge gaps warrant an exploration of the association between demographic and clinical characteristics, including early childhood trauma, childhood sexual abuse, and current stressful life events, and their association with treatment outcome in patients with CD/FND.

## 2 OBJECTIVES

1. Describe demographic and clinical characteristics of patients presenting themselves with CD/FND in a clinical centre of excellence for somatic symptom disorders and related disorders (SSRD) in the specialty mental health setting.
2. Explore the association between predictive factors and treatment outcome in terms of physical symptoms as the primary outcome.
3. Explore the same association with secondary outcomes; depression, anxiety, general functioning, and pain.

## 3 MATERIALS AND METHODS

### 3.1 Design

Prospective longitudinal observational study in a clinical cohort. This study is reported following the STROBE statement (Group S, 2007).

### 3.2 Participants and setting

Consecutive outpatients presenting themselves at the clinical centre of excellence for body mind and health (CLGG), a tertiary specialty mental health setting, with CD/FND between 1 February 2010 and 31 December 2017.

### 3.3 Eligibility criteria

Patients aged 18 years or older, referred to CLGG with CD/FND after neurological assessment and without clear evidence of an underlying somatic condition explaining their symptoms, were eligible. In each case, CD/FND was established by psychiatric examination (PSE), taking all information from the intake into account and according to DSM‐IV criteria (Association AP, 2001) or DSM‐5 criteria (Association AP, 2013), and if needed, including MINI interview.. Eligible patients were identified in the data warehouse of the specialized mental health institution (SMHI). Patient files of eligible patients were checked for consent. Patients were excluded if they did not complete any PROM questionnaires during intake and during treatment; and in case of IQ < 80; or substance dependency.

### 3.4 Variables

This study explores type of CD/FND, comorbid somatic and mental disorders, early childhood trauma and childhood sexual abuse, recent life events, duration of symptoms and of earlier treatment before referral to the clinic, psychosocial factors, and family history as possible factors affecting treatment outcome.

### 3.5 Data sources

Patient files were assessed based on a checklist of potential predictive factors. Data were taken from the files according to a checklist that was put together beforehand, based on a review of the literature for possibly relevant predictive factors. A search was performed in PubMed with the MESH terms ‘Conversion Disorder’ and ‘Prognosis’. This yielded 410 hits, of which, apart from the articles as described in the introduction, three more articles were identified to be of relevance (Jalilianhasanpour et al., 2018; Krishknakumar, Sumesh, & Mathews, 2006; Plioplys et al., 2014; Roelofs et al., 2005). This resulted in the checklist laid down in Table 1.

**Table 1:** Data sources and measurements

Factors	Classification	Based on
Conversion disorder as main diagnosis	Yes No	Patient file: DSM classification (Association AP, 2001) (if unclear, also MINI; Sheehan et al., 1998)
Type of conversion	With sensory symptoms With motor symptoms With nonepileptic seizures or convulsions With mixed symptoms Other	Patient file: intake letter and DSM classification
Development	Acute Gradually	Patient file: intake letter
Time between development of symptoms and treatment in SMHI	<3 months 3–6 months 6–12 months >12 months	Patient file
Time period until treatment in CLGG	Classified in months	Patient file
Psychiatric comorbidity	Yes No Traits of	Patient file: DSM classification and SCID‐2 (Spitzer & Williams, 1983)
Personality disorder	Yes No	Patient file: DSM classification (if unclear, also MINI)
Anxiety disorder	Yes No	Patient file: DSM classification (if unclear, also MINI)
Depressive disorder	Yes No	Patient file: DSM classification (if unclear, also MINI)
Psychotic disorder	Yes No	Patient file: DSM classification (if unclear, also MINI)
Other somatoform disorders	Yes No	Patient file: DSM classification (if unclear, also MINI)
Developmental disorder	Yes No	Patient file: DSM classification (if unclear, also MINI)
Addiction	Yes No	Patient file: DSM classification (if unclear, also MINI)
Somatic comorbidity known as influence in conversion disorders		
Thyroid disease	Yes No	Patient file: DSM classification, intake report (if needed also ICD classification)
Adrenal gland disorder	Yes No	Patient file: DSM classification, intake report (if needed also ICD classification)
Cerebrovascular accident	Yes No	Patient file: DSM classification, intake report (if needed also ICD classification)
Epilepsy	Yes No	Patient file: DSM classification, intake report (if needed also ICD classification)
Mixed image with other neurological disorder	Yes No	Patient file: DSM classification, intake report (if needed also ICD classification)
Other somatic diseases	Yes No	Patient file: DSM classification, intake report (if needed also ICD classification)
Psychosocial factors		
Relationship status	Single Living together Married Living apart together Other	Patient file: intake or registration form Or Psychodiagnostic examination: INTERMED (Huyse et al., 1999)
Family composition	Single without children Single with children With a partner without children With a partner with children	Patient file: intake or registration form Or Psychodiagnostic examination: INTERMED
Social safety net	Good (both contact with friends and family) Moderate (only a single family member or a single friend) Bad (no friends/family)	Patient file: intake or registration form Or Psychodiagnostic examination: INTERMED
Education level	Very low (primary school) Low (VMBO + MBO1, HAVO/VWO junior high school) Medium (HAVO + VWO + MBO 2,3,4) High (HBO‐/WO‐bachelor) Very high (WO‐master + WO‐promotion)	Patient file: intake or registration form Or Psychodiagnostic examination: INTERMED (Huyse et al., 1999)
Work	Employed Sickness law Unemployment benefits Social assistance benefit Rejected (WAO/WIA/IVA) Retired	Patient file: intake or registration form Or Psychodiagnostic examination: INTERMED (Huyse et al., 1999) or LCU (Holmes & Rahe, 1967)
Death of a loved one shortly before the onset of conversion disorder (<6 months)	Yes No	Patient file: intake or registration form Or Psychodiagnostic examination: INTERMED (Huyse et al., 1999) or LCU (Holmes & Rahe, 1967)
Early childhood trauma	Yes No	Patient file: intake and PSE for early childhood trauma and childhood sexual abuse; and ACE (Anda & Felitti, 1998). The ACE International Questionnaire (ACE‐IQ) is developed by the WHO. It is intended to measure ACEs in all countries, and the association between them and risk behaviors in later life. ACE‐IQ is designed for administration to people aged 18 years and older. Questions cover family dysfunction; physical, sexual, and emotional abuse and neglect by parents or caregivers; peer violence; witnessing community violence, and exposure to collective violence. ACE‐IQ is currently being validated through trial implementation as part of broader health surveys (http://www.who.int/violence_injury_prevention/violence/activities/adverse_childhood_experiences/global_research_network/en/). Development has been ongoing and for this study, the available version in 2015 was used. This covers mostly ACE indicating family dysfunction, physical, sexual, and emotional abuse and neglect by parents or caregivers. It was translated from English to Dutch and back‐translated to provide the official Dutch version (Feltz‐Cornelis et al., 2019)
Other life events	Yes No	Patient file: intake and PSE, LCU (Holmes & Rahe, 1967)
Medication use		
Antidepressants	Yes No	Intake report
Benzodiazepines	Yes No	Intake report
Antipsychotics	Yes No	Intake report
Pain relief (except opiates)	Yes No	Intake report
Opiates	Yes No	Intake report
Psychiatric disorders in first‐degree family members		
Conversion disorder	Yes No	Intake report and heteroanamnesis in psychodiagnostic examination
Other disorder	Yes No	Intake report and heteroanamnesis in psychodiagnostic examination

The standard intake procedure at the CLGG consists of questionnaire assessment during intake (referred to as baseline measurement), medical history assessment, physical assessment including neurological examination, psychiatric evaluation, and psychodiagnostic assessment. Throughout treatment, patient's progress was evaluated using a digital PROM (Van der Feltz‐Cornelis et al., 2014). For this study, we used PROM data with regard to physical symptoms, depression and anxiety scores, and pain and general functioning scores.

### 3.6 Data sources/measurements

The variables used and the data sources are shown in the table below. Patient files were checked for the respective measurements. ‘Insert’ Table 1 (Supplementary material).

### 3.7 Treatment

Treatment at CLGG was standardized and multimodal, following the multidisciplinary guideline for medically unexplained symptoms and somatic symptom disorders, with a focus on CD/FND (Van der Feltz‐Cornelis, Hoedeman, Keuter, & Swinkels, 2012; Van der Feltz‐Cornelis, Swinkels, Blankenstein, Hoedeman, & Keuter, 2011). Treatment contained two parallel tracks:

On the one hand, exploring the somatic history and diagnostic assessments carried out leading to the diagnosis of CD/FND; providing explanation of the physical symptoms and psychoeducation to the patient; providing treatment of any relevant identified somatic condition; or revisiting diagnostic considerations while consulting the referring clinician.

On the other hand, providing treatment of the accompanying psychological distress or mental disorders, tailored to the needs and treatment expectations of the patient. Treatment consisted of cognitive behavioral therapy (CBT) (Liu, Gill, Teodorczuk, Li, & Sun, 2019), acceptance and commitment therapy (ACT) (Barrett‐Naylor & Dawson, 2018; Cope & Agrawal, 2017), or problem‐solving treatment (PST) (Malouff, Thorsteinsson, & Schutte, 2007; Wilkinson & Mynors‐Wallis, 1994) provided by trained psychologists, in combination with pharmacotherapy provided by a physician or psychiatrist. The psychotherapeutic treatments were provided sequentially and were tailored to the needs and treatment expectations of the patients. Every 3 months during treatment, both the psychotherapeutic and pharmacotherapeutic treatments were adjusted based on (a) progress in terms of PROM (Black, 1996) and (b) shared decision‐making supported by multidisciplinary team consultation (Van der Feltz‐Cornelis et al., 2014). Patients were treated for 1 year on average, using this multimodal approach.

### 3.8 Bias

Bias was avoided by checking all patient files systematically for the predictive factors according to the checklist which was derived from a review of the literature. In a pilot, the data feeding the checklist were extracted in duplicate (RH and JvE), and the feasibility of this approach was indicated after assessment in 14 files. The data extraction was therefore continued based on this checklist.

### 3.9 Study size

Study size was determined by the number of patients with CD/FND (*N* = 64). Based on this number, we estimated that it would be possible to establish an association between treatment outcome and 6 predictive factors with sufficient power (Nunnaly & Bernstein, 1994).

### 3.10 Quantitative variables

Primary outcomes were physical symptoms measured by the Patient Health Questionnaire (PHQ15) and the Physical Symptoms Questionnaire (PSQ) (Van Hemert, 2003) at end of treatment. Secondary outcomes were anxiety, depression, pain, and general functioning at end of treatment measured by the Patient Health Questionnaire for Depression (PHQ9) (Kroenke et al., 2009) and Generalized Anxiety Disorder (GAD7) (Kroenke, Spitzer, Williams, & Löwe, 2010), the Brief Pain Inventory (BPI) (Cleeland & Ryan, 2004), and Short Form 36 (SF‐36) (Ware & Sherbourne, 1992) as indicated in Table 1.

Covariates were type of CD/FND, childhood adverse experiences (ACE) including childhood sexual abuse, life events, comorbid anxiety disorder, somatoform disorder, depressive disorder or personality disorder and developmental disorder, comorbid somatic disorder, treatment history, age, gender, family history, and civic status. Dichotomous variables were coded at 1 (yes) and 0 (no). Dummy variables were produced for >2 categories (e.g., personality disorder) and entered into a model together.

### 3.11 Statistical methods

Frequencies were explored to establish prevalence of the prognostic factors in the sample. Linear regression analyses using enter method were performed to explore associations of predictors with treatment outcome in CD/FND. Analyses were performed using listwise deletion to address cases of missing data. For the secondary outcomes, hierarchical multiple regression analyses were performed.

### 3.12 Sensitivity analyses

One sensitivity analysis explored the associations while controlling for baseline scores of secondary outcomes. Another sensitivity analysis explored if patients following up the intake with treatment at the CLGG differed significantly from patients who did not proceed with treatment in terms of baseline characteristics.

## 4 RESULTS

### 4.1 Sample characteristics

**Figure 1:**
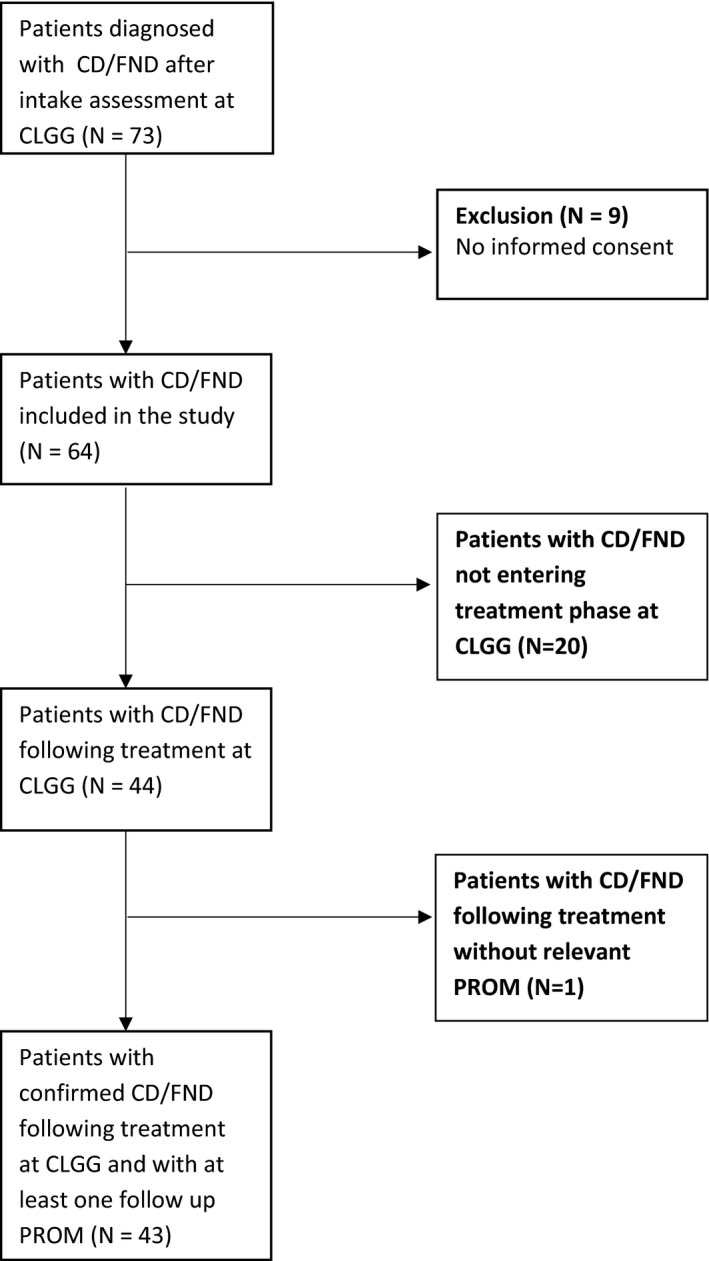
Flowchart participants

Seventy‐three consecutive patients were diagnosed with CD/FND at intake in CLGG. Nine patients declined use of their patient file data for scientific research, and their data were not included. Of the 64 remaining patients, 20 patients did not enter treatment at CLGG. Forty‐four patients followed treatment at CLGG, 43 of which filled at least one follow‐up PROM. Patient flow is shown in the flow chart (Figure 1).

Demographic and clinical characteristics of the 64 patients with CD/FND are shown in Table 2.

**Table 2:** Demographic and clinical characteristics of conversion disorder/functional neurological disorder patients (*N* = 64)

	CD/FND (*n* = 64)			CD/FND (*n* = 64)	
	*n*	%		*n*	%
Sex			Type of Conversion disorder		
Male	13	20.3	With sensoric symptoms	5	7.8
Female	51	79.5	With motor symptoms	25	39.1
			With nonepileptic seizures	9	14.1
Age	*M* = 43.14	*SD* = 11.58	With mixed symptoms	17	26.6
			Other	6	9.4
Relationship status					
Single	21	32.8	Time between symptom onset to start of treatment		
Cohabiting	14	21.9	<3 months	5	7.8
Married	24	37.5	3–6 months	6	9.4
Long‐distance	5	7.8	6–12 months	10	15.6
			>12 months	42	65.6
Family status					
Single no children	20	31.3	Onset		
Single with children	7	10.9	Acute	27	42.2
Partner no children	10	15.6	Gradually	37	57.8
Partner with children	27	42.2			
			Time from symptom onset to start of treatment in CCLG (months)	*M* = 61.11	*SD* = 70.49
Social network					
Good	27	42.2	Comorbid disorders		
Mediocre	31	48.4	Personality disorder	26	40.6
Bad	5	7.8	Anxiety disorder	31	48.4
			Depressive disorder	27	42.2
Education			Psychotic disorder	2	3.1
Very low	8	12.5	Developmental disorder	11	17.2
Low	20	31.3	Addiction	3	4.7
Middle	23	35.9	Thyroid disorder	7	10.9
High	9	14.1	Adrenal disorder	0	0
Very high	1	1.6	Other somatic disorder	17	26.6
			Stroke	7	10.9
Work status			Epilepsy	2	3.1
Working	13	20.3	Other neurological condition	6	9.4
Sickness benefits	15	23.4	Other somatic condition	40	62.5
Unemployment benefits	4	6.3			
Social assistance benefit	7	10.9	Use of Medication		
Disabled	18	28.1	Antidepressants	29	45.3
Retired	2	3.1	Benzodiazepines	17	26.6
			Antipsychotics	5	7.8
Trauma/Stress			Pain medication	24	37.5
Childhood trauma	45	70.3	Opiates	12	18.8
Recent life event	41	64.1			
Sexual abuse in childhood	17	26.6	Pain (*n* = 62)		
Death of a loved one	3	4.7	BPI > 0	57	91.9
Adverse childhood events (ACE) (*n* = 30)			BPI ≥ 3	54	87.1
ACE > 0	24	80	Mean BPI score	*M* = 5.09	*SD* = 3.39
ACE > 1	22	73.3			
ACE ≥ 4	13	43.3			
Mean ACE scores	*M* = 4.10	*SD* = 3.78			
Family history					
Family member with CD/FND	0	0			
Family member with other psychiatric disorder	24	37.5			

Mean age was 43 years. About 79.5% of the sample were female, and 59.4% were married or living together. The majority of the patients had a history of multiple diagnostic procedures by neurologists and other medical specialists and started psychiatric treatment at least 12 months after the onset of the CD/FND symptoms. On average, 5 years elapsed before presentation at CLGG. The most common CD/FND was with motor symptoms (39.1%). CD/FND with mixed symptoms came second (26.6%). One patient suffered from foreign accent syndrome, which, if of functional nature, can be considered a conversion disorder (Keulen et al., 2016). About 66% started psychiatric treatment more than 12 months after onset of the CD/FND symptoms.

In the whole sample, 70.3% reported childhood trauma. In the participants who filled in the ACE (*N* = 30), 80% reported at least one ACE, 73.3% reported more than one ACE, and 43.3% reported an ACE score of 4 or more. The average ACE score was 4.10 (*SD* 3.78). Sexual abuse in childhood was reported by 26.6% of the patients, recent life events by 64.1%, and recent death of a loved one by 4.7%.

In the whole sample, 87.1% of the patients reported concomitant chronic pain. In the participants who filled in the BPI (*N* = 62), 91.9% of the patients reported pain, and 87.1% presented with a baseline score of 3 or more on a scale of 0–10, which is considered as pain of clinical significance (Brannan et al., 2005; Brecht et al., 2007; Tan, Jensen, Thornby, & Shanti, 2004). Pain requiring pain medication including opiates occurred in 56.3% of the sample.

### 4.2 Treatment outcome

Table 3 shows the mean scores for the primary outcomes (PSQ) and secondary outcomes at baseline and end of treatment in patients with CD/FND.

**Table 3:** Paired samples *t* test indicating improvement in primary and secondary outcomes from baseline to end of treatment in group who received treatment (*n* = 43)

Outcome	Baseline (*M* [*SD*])	End of treatment (*M* [*SD*)	*t*	*df*	*p*
PSQ (*n* = 30)	23.33 [21.54]	17.70 [22.23]	2.022	29	**.**052
PHQ15 (*n* = 28)	12.25[5.30]	10.00 [5.31]	2.294	27	**.030** *
PHQ9(*n* = 41)	13.63 [7.16]	10.88 [8.22]	2.355	40	**.023** *
GAD7(*n* = 35)	10.51 [5.24]	6.97 [5.37]	3.545	24	**.001** **
SF36(*n* = 26)	17.54 [3.84]	15.88 [3.36]	2.006	25	.056
BPI(*n* = 38)	5.68 [2.60]	5.58 [2.51]	0.246	37	.807

Note

Missing data (*n*) for the 43 patients who completed at least one follow‐up PROM was PSQ = 1 (2.3%); PHQ15 = 1 (2.3%);GAD7 = 2 (4.7%); SF36 = 9 (20.9%) at baseline; and PSQ = 13 (30.2%); PHQ15 = 15(34.9%); PHQ9 = 2 (4.7%); GAD7 = 7 (16.3%); SF36 = 14 (32.6%); and BPI = 5 (11.6%) at follow‐up.

* *p *< .05 (Mean scores [*SD*]).

** *p *< .001.

Bold indicates significant finding.

Paired samples *t* tests show that physical symptom scores (PHQ15), depressive symptoms (PHQ9), and anxiety symptoms (GAD7) decreased significantly from baseline to end of treatment. The decrease in PSQ, another questionnaire exploring physical outcomes, and general functioning (SF36) approached significance (*p *= .052 and *p *= .056, respectively). Pain symptoms (BPI) did not show a significant change.

In Table 4, differences in outcomes are shown between patients who experienced childhood sexual abuse and those who did not.

**Table 4:** Independent samples *t* tests indicating differences in outcomes between patients who had encountered sexual abuse in childhood and those who had not within the group who received treatment (*n* = 43)

Outcome (*M* [*SD*])		Childhood sexual abuse		*t*	*df*	*p*
		Yes (*n* = 11)	No (*n* = 32)			
PSQ	Baseline	35.36 [26.56]	22.34 [21.43]	1.635	41	.110
	Follow‐up	39.75 [33.08]	9.68 [9.00]	4.054	28	**.000** **
PHQ15	Baseline	13.00 [4.60]	14.25 [6.35]	−0.599	41	.552
	Follow‐up	10.83 [5.31]	9.77 [5.42]	0.427	26	.673
PHQ9	Baseline	14.82 [7.15]	13.52 [7.08]	0.528	42	.601
	Follow‐up	14.78 [8.59]	9.78 [7.90]	1.646	39	.108
GAD7	Baseline	12.00 [6.18]	10.71 [5.25]	0.669	40	.507
	Follow‐up	10.33 [6.82]	6.15 [4.61]	2.086	34	**.045** *
SF36	Baseline	18.43 [3.78]	17.61 [3.96]	0.495	33	.624
	Follow‐up	16.20 [3.56]	15.21 [4.15]	0.496	27	.624
BPI	Baseline	5.09 [3.39]	5.52 [2.43]	−0.454	42	.652
	Follow‐up	6.30 [1.25]	5.32 [2.80]	1.060	36	.296

* *p *< .05 (mean scores, *SD*).

** *p *< .001.

Bold indicates significant finding.

Independent samples *t* tests show that at the end of treatment those who suffered sexual abuse in childhood reported significantly higher scores on the PSQ (physical symptoms) and on the GAD7 (anxiety) than those who did not report sexual abuse in childhood.

#### 4.2.1 Predictors of treatment outcome

Significant associations between predictors and primary outcomes and between predictors and secondary outcomes are shown in Table 5.

**Table 5:** Linear regression analyses showing significant associations between predictors and primary (PSQ) and secondary outcomes

Predictor	Outcome	*b*	*SE* *b*	*β*	*t*	*p*	*R* ^2^
Primary outcomes							
Sexual abuse in childhood	PSQ	30.068	7.417	0.608	4.054	**.001** **	.370
Secondary outcomes							
Depressive disorder	PHQ9	7.755	2.284	0.478	3.395	**.002** *	.228
Depressive disorder	GAD7	4.712	1.671	0.435	2.820	**.008** *	.190
Developmental disorder	BPI	−2.636	0.970	−0.412	−2.716	**.010** *	.170

Note

*b* = unstandardized beta, *SE b* = standard error of unstandardized beta, *β* = standardized beta, *R* ^2^ = *R*‐squared.

* *p* < .05.

** *p* < .01.

Bold indicates significant finding.

With respect to the primary outcomes, linear regression analyses indicated sexual abuse in childhood (*F*[1, 28] = 16.435, *β* = 0.608, *p *< .001) was the only significant predictor of physical symptoms as measured by the PSQ at follow‐up. The presence of ACE and having an ACE score of 4 or more were not significantly associated with treatment outcome, and neither was the presence of current stress. In terms of secondary outcomes, linear regression analyses indicated that the presence of a comorbid depressive disorder at intake was significantly associated with higher scores on the PHQ9 (*F*[1, 39] = 11.526, *β* = 0.478, *p *= .002) and GAD7 (*F*[1, 34] = 7.950, *β* = 0.435, *p *= .008) at follow‐up. A comorbid developmental disorder such as adult ADHD or autism spectrum disorder was significantly associated with lower BPI (*F*(1, 37) = 7.379, *β* = −0.412, *p *= .010) scores at follow‐up. All other associations, including experiencing a recent major life event, were nonsignificant.

#### 4.2.2 Sensitivity analyses

Hierarchical multiple regression analyses showed that when baseline scores were controlled for, comorbid depressive disorder remained a significant predictor of both the PHQ9 (*β* = 0.324, Δ*R* ^2^ = .090, *p *= .025) and GAD7 (*β* = 0.328, Δ*R* ^2^ = .099, *p *= .049) scores at follow‐up within each respective model. Also, when baseline BPI scores were entered into the model, the presence of a comorbid developmental disorder was not a significant predictor of BPI scores at follow‐up (*β* = −0.263, Δ*R* ^2^ = .037, *p *= .106).

Another sensitivity analysis explored if patients following up the intake with treatment at the CLGG differed significantly from patients who did not proceed with treatment in terms of baseline characteristics. The only significant difference was found for anxiety symptoms, with mean baseline GAD7 score of 6.56 (*SD* = 5.45) in the no‐treatment group versus 11.05 (*SD* = 5.46) in the treatment group (*X* ^2^ = 6.396 (1), *p *= .011).

## 5 DISCUSSION/CONCLUSION

### 5.1 Summary of principal findings

The majority of the patients had a history of multiple diagnostic procedures by neurologists and other medical specialists, and started psychiatric treatment more than 12 months after onset of the CD/FND symptoms. On average, 5 years elapsed before presentation at CLGG, which is a specialized center providing treatment to the top 5% most complex cases of CD/FND and other SSRD (van Eck van der Sluijs, de Vroege, van Manen, Rijnders, & van der Feltz‐Cornelis, 2017). Hence, this sample can be considered as a sample of patients with chronic CD/FND referred to a tertiary care specialized mental Health Institution for SSRD.

The percentage of patients reporting at least one ACE in this study, eighty percent, is higher than the 24%–50% reported in earlier research in CD/FND (Régny & Cathébras, 2016; Roelofs et al., 2005; Selkirk et al., 2008) and higher than the 64% reported in the original ACE field study among 17,000 people visiting a medical evaluation center in the USA (Centers for Disease Control & Prevention, 2016). This is also higher than the 77.2% of patients reporting at least one ACE in a study in outpatients with depressive or anxiety disorders in another department of the same specialty mental health institution, conducted by this research group (Feltz‐Cornelis et al., 2019). The percentage of patients reporting more than one ACE (73%), the mean ACE score of more than 4, and the percentage reporting a score of 4 or more (43%) are higher as well. An ACE score of 4 or more has been found to be associated with depressive disorders, suicide attempts, and alcohol abuse (Anda & Felitti, 1998; Dube, Felitti, Edwards, & Croft, 2002; Edwards, Felitti, & Anda, 2003). Therefore, based on the findings of this study, indications are that CD/FND can join this list of mental disorders associated with high levels of adverse childhood experiences.

Recent life events are highly prevalent as well; however, although the burden of general ACE and recent stressful events in this study is high, they are not associated with treatment outcomes. The primary and several secondary treatment outcomes improved significantly after treatment in the whole group and the only predictor significantly associated with worse physical treatment outcome was childhood sexual abuse. A separate analysis shows that not only worse physical outcomes but also higher anxiety levels at end of treatment in those who suffered sexual abuse in childhood.

Comorbid depressive disorder has a significant negative association with secondary treatment outcome in terms of depressive and anxiety symptoms. This might be interpreted, as that CD/FND is harder to treat whether comorbid depressive or anxious disorder feed into catastrophic interpretations of the symptoms and the expected course.

Pain seems to be a physical symptom of particular importance in patients with CD/FND as clinically significant levels of pain occur in 87.1% of the sample, and pain requiring medication occurs in more than 55% of the sample. Moreover, the mean BPI score is over 5, which is higher than the mean score reported in a study on cancer patients with bone metastases (Zeng et al., 2011). Stone and Sharpe find a high prevalence of pain in functional weakness (Stone, Warlow, & Sharpe, 2010), and possible interpretations of the association between chronic regional pain syndrome and CD/FND have been discussed (Popkirov, Hoeritzauer, Colvin, Carson, & Stone, 2019). However, so far, pain has not received much attention in CD/FND research, classification, and guidelines. In this study, pain did not improve in the group as a whole, but did improve significantly in case of comorbid developmental disorders such as adult ADHD or autism spectrum disorder. This influence on treatment outcomes disappeared after correction for baseline pain levels, indicating that patients with adult ADHD or autism spectrum disorder had higher pain levels at intake, and benefitted from the treatment for pain outcomes. This patient group benefitted relatively better from the treatment intervention, at least for the pain component of their condition, than the group as a whole.

The sensitivity analysis comparing the patients that continued to treatment with the patients that did not showed a significant difference in anxiety, with level of anxiety amounting to the level of anxiety disorder (GAD score 10 or more) in the treatment group versus far below this threshold in the no‐treatment group. From a clinical point of view, this makes sense as it can be interpreted as those with higher levels of anxiety have a higher motivation for treatment and could also reflect levels of health anxiety related to the symptoms for which treatment was sought.

### 5.2 Strengths and weaknesses of the study

A strength of this study is the longitudinal, prognostic design, and the exploration of predictors of treatment outcome, including childhood sexual abuse and psychiatric comorbidity, which is a first in the literature. This way we could establish for the first time that sexual abuse in childhood is associated with worse physical treatment outcome; that comorbid depression has a negative impact on concomitant depressive and anxiety symptoms in CD/FND; and that patients with comorbid CD/FND, pain, and comorbid developmental disorder benefit more from treatment. The fact that this study is conducted in a specialty mental health setting can also be seen as a strength of the study, as so far, studies have been performed in neurology clinics that in general see first presentations, whereas here we have the opportunity to explore a cohort with chronic CD/FND. Another strength of the study is that we were able to perform sensitivity analyses showing our findings to be robust and indicating possibly higher health anxiety levels in the group proceeding to treatment at CLGG.

Limitations of the study are that due to the study setting and patient population, the findings of this study are generalizable to chronic complex CD/FND treated in the specialty mental health setting, but not to patients with CD/FND that present themselves for the first time in neurology clinic. Another limitation is that although in comparison with other studies in this field, the sample size of this study can be considered large, it is relatively small compared to other clinical epidemiological cohort studies.

### 5.3 Implications

This study shows that treatment involving a parallel track combination of revisiting the somatic history, providing psychoeducation, and providing tailored psychotherapeutic and medical treatment in a shared decision model (SDM) (Van der Feltz‐Cornelis et al., 2014) is associated with significant improvement of somatic and psychological treatment outcomes. As this was an observational study, and in view of the lack of effective evidence‐based treatments for this condition, a randomized clinical trial exploring treatment effect of the described intervention is warranted.

The finding that childhood sexual abuse is associated with significantly worse treatment outcome warrants the development and evaluation of treatment interventions specifically targeting childhood sexual abuse in CD/FND alongside regular treatment.

Furthermore, the high prevalence of pain in this study, and the finding that pain did not improve in the group as a whole, warrants development of treatment models focusing at pain in CD/FND. The susceptibility of pain to improvement in patients with comorbid adult developmental disorders warrants development and evaluation of medical and psychotherapeutic treatment models specifically targeting pain in CD/FND with comorbid adult ADHD/autism spectrum disorder, as this may be a subgroup of patients with potential for more benefit of treatment.

## AUTHOR CONTRIBUTIONS

CFC designed the study, facilitated the conduct of the study, oversaw the analysis, and wrote the paper. SFA codesigned the study, designed the analysis, contributed to interpretation of the analysis, and coauthored the paper. JvEvdS Codesigned the study, oversaw data extraction, contributed to interpretation of the analysis, and coauthored the paper. All authors approved the final version of the manuscript.

## ETHICAL APPROVAL

This study follows the principles of the World Medical Association's Declaration of Helsinki. No informed consent was required, as for the present research we used data that were collected for administrative purposes and monitoring of treatment outcome by treatment providers. According to Dutch law, in accordance with the Helsinki Declaration, and according to the Dutch Central Medical Ethical Committee, no explicit informed consent is required for the use of clinical or administrative data, collected in the context of treatment provision. At intake, patients were informed that patient‐reported outcome measures (PROM) and medical data obtained during intake and treatment could be used for research evaluation on an anonymous basis, unless they indicated their dissent. In such cases, this was notified in the patient file. Patient files of dissenting patients were excluded from the study. Data were coded in order to create an anonymous dataset. The research protocol was approved by the IRB scientific committee of GGz Breburg (2017–03).

## Data Availability

The data are owned by a third party, GGz Breburg, that does not publicly share data. However, interested parties will be able to obtain data upon request as follows. Researchers can submit a research plan, which describes the background and methods of a proposed research question, and a request for specific data of the database used for this study to answer the research question. After approval of the research plan by the principal investigator and the director of GGz Breburg, a deidentified minimal dataset can be obtained. Information can be requested by contacting the principal investigator: Dr. Jonna van Eck van der Sluijs, email: j.vanEckvanderSluijs@ggzbreburg.nl.
